# A call for action to stop sexual violence against women in the Democratic Republic of Congo: A brief report

**DOI:** 10.1002/ijgo.15801

**Published:** 2024-08-01

**Authors:** Olivier Nyakio, Denis Mukwege, Fabien Balagizi, Malik Olatunde Oduoye, Styves Banga, Jones Onesime, Priyadarshini Bhattacharjee, Hardy Elembwe, Hugues Cakwira, Elie Kihanduka, Afissa Amiri, Excellent Rugendabanga, Samson Hangi, Chloe Makungu, Aymar Akilimali

**Affiliations:** ^1^ Faculty of Medicine Evangelic University in Africa Bukavu Democratic Republic of the Congo; ^2^ Faculty of Medicine Official University of Bukavu Bukavu Democratic Republic of the Congo; ^3^ Department of Research Medical Research Circle Bukavu Democratic Republic of the Congo; ^4^ Standing Committee of Research and Exchange Medical Student Association Bukavu Democratic Republic of the Congo; ^5^ Society of Maternal‐Fetal Medicine Washington District of Columbia USA; ^6^ Department of Clinical Medicine, Cambridge University Hospitals NHS Foundation Trust University of Cambridge Cambridge UK; ^7^ Faculty of Medicine Université Libre des Pays des Grands Lacs Goma Democratic Republic of the Congo; ^8^ Faculty of Medicine La Sapientia Catholic University Goma Democratic Republic of the Congo

**Keywords:** adolescent, adult, Democratic Republic of Congo, female, sexual violence, stop, women

## Abstract

Sexual violence in the Democratic Republic of Congo is also described as a weapon of war.

Sexual violence against women is a human rights violation and a major public health problem, with far‐reaching consequences for women's health.^1^ Sexual violence against women (especially young women and children) has become an epidemic in the Democratic Republic of Congo (DR Congo), although literature on the issue is lacking. The frequency of sexual violence is particularly high in the eastern part of the country due to the armed conflicts that reign in this region.^2^ The DR Congo is considered “the rape capital of the world” due to the high prevalence and intensity of all kinds and forms of sexual violence against women and children, described as the worst in the world.^3^ Massive and collective sexual violence, sexual violence in public, sexual slavery, forced incest, evisceration, forced insertion of objects (tree branches and other objects) into the vagina or orifices of victims, sexual violence in front of the victim's family members, the pouring of rubber and other melted plastic objects into women's vaginas, the firing of firearms into women's vaginas, and sexual and genital mutilation are also observed and occur during periods of war against the civilian population in DR Congo.^2^, ^4^ Sexual violence in DR Congo is also described as a “weapon of war,” in accordance with the official United Nations declaration of 2008, and is responsible for post‐traumatic stress disorder.^4^ The world's media must focus on sexual violence as a weapon of war in DR Congo, highlighting the neglected aspect of sexual violence against children and young women.^2^ There are limited reports and studies on sexual violence against women there have been few campaigns focused on sexual violence against women in DR Congo. Given the lack of published studies on the issue, we have written the present article to highlight the prevalence, burden, and risk factors of sexual violence against women in DR Congo as well as to discuss possible solutions to put an end to the problem.

The number of Congolese affected by sexual violence has been increasing in DR Congo. Unfortunately, there is limited data on the actual prevalence of sexual violence in DR Congo. However, the Office for the Coordination of Humanitarian Affairs reported that between 2021 and 2022, the number of reported cases of gender‐based violence in DR Congo rose from 40 000 to over 80 000 victims. This year alone, precisely in the first 3 months of 2023, over 31 000 cases of gender‐based violence were reported.^5^ Women who have suffered sexual violence are victims of physical assault and injury (these physical injuries vary in severity from abrasions and contusions to concussions, fractures, and gunshot wounds) and mental health problems such as post‐traumatic stress. Sexual violence is not limited to those experiencing poverty, internal displacement, and lack of education.^5^ Other factors that could predispose a woman to sexual violence include promiscuity and dressing indecently, which could lead to sexual attraction by the perpetrator (even though this should not justify this sexual immorality), peer pressure, drug abuse, smoking, and alcohol consumption.

The major consequences of sexual assault on women's health are genital lesions and injuries, sexually transmitted infections, and unwanted pregnancies.^6^ Therefore, we recommend that non‐governmental organizations, such as UNICEF and Médecins Sans Frontières, intensify their activities to prevent and respond to sexual violence, providing essential medical and psychosocial services to girls and women affected by sexual violence in the province of North Kivu, in the eastern region of DR Congo. There should be increased efforts at psychotherapeutic rehabilitation such victims of sexual abuse.

The Congolese Government and its Ministry of Health must provide medical care equipment and build reception services and care centers for female survivors of sexual violence in all regions, especially in the most remote areas of the country, where the incidence of sexual violence is very high. Awareness of the problem and the consequences of sexual violence in the country must motivate national and international humanitarian and health actions to prevent the continuation of the atrocities committed.

According to Margot Wallström, DR Congo is one of the most dangerous places on Earth for a woman, and rape is simply a fact of life in the country.^7^ The number of victims of sexual violence living in DR Congo today is estimated at 200 000. The United Nations Population Fund has reported that over 65% of victims are children (the majority of whom are adolescents).^7^ In the province of North Kivu, where armed conflict is rife, the frequency of sexual violence increased by 37% in the first 3 months of 2023.^8^ To reduce this very high frequency and avoid the worst in the coming years, we recommend that UNICEF (a charity working in this region) intensify its activities to prevent and respond to sexual violence, providing essential medical and psychosocial services to girls and women affected by sexual violence in North Kivu.

To put an end to sexual violence in DR Congo, we are calling on the Congolese Government in collaboration with international human rights organizations to put in place strict and abiding laws that strongly condemn the perpetrators of crimes of sexual violence. These laws must be enacted to protect women from sexual violence. The Congolese Government and its Ministry of Justice must personally investigate the perpetrators of crimes of sexual violence and set up new courts to prosecute those accused of sexual violence. We call on the Congolese Government, as well as local authorities in regions in DR Congo affected by sexual violence, partners, and donors, to take all necessary measures to put an immediate end to sexual violence against women and to protect women and girls who have already been victims of displacement due to sexual violence.

Furthermore, the Congolese Government should collaborate with the UNFPA to create increased awareness about sexual violence against women in DR Congo. This awareness should include both men and women, children, and young and older adults in the country and should be well constructed and specific, pointing out the detrimental effects of sexual violence. The UNFPA should also deploy more representatives to DR Congo to provide better sexual health and reproductive health services for the Congolese population, especially those that are vulnerable and victims of sexual violence.

Religious and traditional leaders also have a role to play in ending sexual violence in the DR Congo. Religious and traditional leaders are crucial to solving health issues in the community, especially during religious and traditional activities. Daily sermons could include messages condemning sexual violence in DR Congo. We also urge all educational authorities in DR Congo to include sexual education in the curricula of all schools in the country. Through this initiative, pupils in primary and secondary schools as well as students in tertiary institutions would have good knowledge, attitudes and perceptions of sexual violence and its preventive measures. Healthcare providers in DR Congo also have work to do in putting an end to sexual violence in DR Congo by creating greater awareness about sexual violence among all patients coming to receive treatment in the healthcare facilities in the country. An adage says that charity begins at home. Parents and guardians in DR Congo need to train their children properly, telling them to stay away from bad friends and illicit behaviors, like drug abuse, smoking, alcohol consumption, and other social vices, as these are some of the predisposing factors of sexual violence. We also recommend more Knowledge, Attitude and Practices surveys and research on sexual violence be conducted by students, doctors, and researchers in DR Congo.

In conclusion, sexual violence against women in DR Congo requires all hands on deck to put an end to the menace of sexual violence in the country.

## AUTHOR CONTRIBUTIONS

*Conception*: Aymar Akilimali and Malik Olatunde Oduoye, *Design*: Malik Olatunde Oduoye, *Project administration*: Aymar Akilimali, *Supervisor*: Olivier Nyakio, *Funding acquisition*: Olivier NYAKIO, *Investigation*: Malik Olatunde Oduoye, *Resources*: Aymar Akilimali and Fabien Balagizi, *Literature search*: All Authors, *Manuscript preparation*: All Authors, *Manuscript editing*: Malik Olatunde Oduoye, Hugues Cakwira and Aymar Akilimali, *Manuscript review*: All authors, *Final approval of manuscript*: All authors.

## FUNDING INFORMATION

The authors did not receive any financial support for this work. No funding has been received for the conduct of this study.

## CONFLICT OF INTEREST STATEMENT

The authors declare that there is no conflict of interest.

## Data Availability

Research data are not shared.
